# A retrospective study of the clinical benefit from acetylsalicylic acid desensitization in patients with nasal polyposis and asthma

**DOI:** 10.1186/s13223-014-0064-7

**Published:** 2014-12-11

**Authors:** Christine Ibrahim, Kulraj Singh, Gina Tsai, David Huang, Jorge Mazza, Brian Rotenberg, Harold Kim, David William Moote

**Affiliations:** Schulich School of Medicine and Dentistry, London, Ontario Canada; Department of Allergy and Immunology, London, Ontario Canada; Department of Otolaryngology, London, Ontario Canada

**Keywords:** Aspirin desensitization, Nasal polyposis, Rhinosinusitis, Asthma, Aspirin-exacerbated respiratory disease

## Abstract

**Background:**

Aspirin-exacerbated respiratory disease (AERD), also known as Samter’s triad, is a clinical syndrome which consists of aspirin (ASA) intolerance, chronic rhinosinusitis with nasal polyposis, and intrinsic bronchial asthma (Press Med 119:48-51, 1922). ASA challenge is the gold standard for diagnosing AERD (Curr Allergy Asthma 9:155-163, 2009). The practice of ASA challenge and desensitization in Canada is infrequently utilized, which may explain its omission as a viable therapeutic option in the latest Canadian clinical practice guidelines for acute and chronic rhinosinusitis (AACI 7:1-38, 2011).

**Methods:**

This retrospective study assessed 111 patients who underwent ASA desensitization in the Allergy and Immunology clinic at St. Joseph’s Healthcare (SJHC) in London, Ontario. The mean age was 50.7 years, and 52.5% (n = 58) were male. Sixty-one percent (n = 68) claimed prior, significant reactions to ASA, and all patients had features of AERD.

**Results:**

Seventy-three percent (n = 81) claimed symptom improvement after achieving maintenance dosing on the desensitization protocol. Of this population, 21.6% (n = 24) improved in all 3 areas of interest (sense of taste or smell, upper respiratory symptoms and lower respiratory symptoms). Twenty-six percent (n = 29) had adverse effects, mostly in the way of gastrointestinal upset, but no severe adverse events were seen.

**Conclusions:**

ASA desensitization helps improve symptoms in patients with AERD. Further, it allows patients to tolerate additional ASA and other non-steroidal anti-inflammatories (NSAIDs) when needed for supplemental analgesia or for cardio-protection. This is of particular benefit in those who require these medications for improved quality of life, and for reduced morbidity and mortality, such as those with cardiovascular disease or chronic pain. There should be further studies conducted in Canada as well as consideration for ASA desensitization to be included in the next clinical practice guidelines.

## Background

Aspirin-exacerbated respiratory disease (AERD) is a clinical syndrome which consists of aspirin intolerance, chronic rhinosinusitis with nasal polyposis and intrinsic bronchial asthma as first described by Widal in 1922 [1]. Max Samter, an American immunologist, revisited the association and proposed the possible pathogenesis in the 1960s. His name is often associated with the syndrome—Samter’s triad [2]. AERD affects 0.3-0.9% of the general population, but its prevalence rises to 10-20% in asthmatics, and up to 30-40% in asthmatics with nasal polyposis [3,4]. Clinical features include onset of nasal congestion with anosmia, with progression to chronic pansinusitis and nasal polyposis. The nasal polyps often re-grow rapidly after repeated surgeries [5]. Asthma may precede the upper airway disease or develop later.

ASA challenge is the gold standard for diagnosing AERD [5]. Zeiss and Lockey were the first authors to describe a 72-hour refractory period after oral ASA challenge in ASA-sensitive patients in 1976 [6]. Since then, multiple studies have shown that desensitization and daily treatment with aspirin can not only allow the medication to be tolerated, but can significantly improve overall symptoms and quality of life, decrease formation of nasal polyps and sinus infections, reduce the need for oral corticosteroids and sinus surgery, and improve nasal and asthma scores in patient with AERD. The effects are noticeable as early as 4 weeks following desensitization [7], and persist at least up to 5 years in to follow-up [8]. Much of what we know about ASA challenge and desensitization derives from studies of over 1400 patients who have undergone the procedure at Scripps Clinic in San Diego, CA, USA. ASA challenge and desensitization has received little attention in Canada, which may explain its omission as a viable therapeutic option in the latest Canadian clinical practice guidelines for acute and chronic rhinosinusitis [9].

Since the use of aspirin desensitization was first described in 1984, and shown to clinically improve the underlying inflammatory airway disease [10], much research has been done to further optimize this procedure. Premedication with leukotriene receptor antagonists, alone or in combination with inhaled corticosteroids and long-acting β2-agonists, was able to reduce lower respiratory tract reactions during aspirin challenge in some patients, but did not change the overall rate of positive aspirin challenge and desensitization [11,12].

The aim of this study was two-fold. First, to assess patient-specific improvement scores and address questions surrounding patient discontinuation. Second, to assess the severity of adverse effects from high-dose ASA maintenance therapy.

## Methods

### Study population

This retrospective study of 111 patients took place at the Allergy and Immunology clinic at St. Joseph’s Healthcare (SJHC) in London, Ontario, from 2007–2011.

Inclusion criteria involved patients seen in consultation at the Allergy and Immunology clinic, an adult (≥ age of 17), who had nasal polyposis, asthma, and a history of ASA or non-steroidal anti-inflammatory (NSAIDs) sensitivity, and had a stable clinical course. They must have then completed a trial of ASA desensitization and attended follow-up for at least one year after. Baseline medical illness and therapies were assessed (Table 1). The use of steroids and antibiotics related to AERD were determined (Table 2).

**Table 1:** **Baseline characteristics of patients**

*Age-year, mean (range)*	50.7 (17–75)	
Male, n (%)	58 (52.2)	
Female, n (%)	53 (47.8%)	
*Length of upper respiratory disease (%)*		
<5 years	22.2	
5-10 years	28.3	
10-15 years	15.2	
>15 years	34.3	
*Length of lower respiratory disease (%)*		
<5 years	33.7	
5-10 years	25.3	
10-15 years	10.8	
>15 years	30.2	
*Baseline FEV1 (n)*		*Post ASA FEV1 (n)*
Mild (≥80)	25	32
Moderate (<80 × ≥50)	36	36
Severe (<50)	2	2
Unknown	48	41

**Table 2:** **Indications of disease severity**

*Prednisone use in past 12 months (%)*	63.80%
*Antibiotic use in past 12 months (%)*	22.90%
*Number of endoscopic sinus surgeries (%)*	
None	11.4
1 to 3	65.7
4 to 6	19.1
7 to 10	1.9
>10	1.9
*Lund-MacKay CT Score (%, from n = 72)*	
0-6	2.6
7-12	13.2
13-18	35.5
19-24	48.7

Subjects were excluded if they had significant concomitant disease, history of life-threatening ASA or NSAID reactions, or other chronic conditions or treatments that may have confounded the interpretation of the study results.

Ethics approval was received from Western University in London, Ontario. Informed consent was obtained, and clinical records were reviewed, looking specifically for ASA desensitization over a two-day protocol. This previously validated protocol is from the Scripps Clinic, with an initial dose of 40 mg titrated up to 162 mg on day one, and then 325 mg on day two [3,7,8]. From past literature, maintenance dose is kept at 325 mg or 650 mg twice daily [3,8,13].

### Patient characteristics

Patient characteristics prior to ASA challenge and desensitization were obtained, including age, duration of upper and/or lower respiratory disease, and baseline percent predicted FEV1 (Table 1). Further severity of disease was assessed based on number of prednisone bursts and antibiotic treatment in the preceding 12 months to desensitization, current asthma and rhinitis controller medications, number of endoscopic sinus operations, and severity of disease on imaging (Tables 2 and 3).

**Table 3:** **Asthma and rhinitis medications**

*Skin test positivity (%)*		63.8	
*Baseline asthma therapy (%)*		*Post-ASA asthma therapy (%)*	
SABA	1.9	SABA	2.7
ICS	2.9	ICS	3.6
LABA	0.0	LABA	0.0
Leukotriene	1.9	Leukotriene	4.5
Combination	80.0	Combination	60.4
Unknown	13.3	Unknown	28.8
*Baseline rhinitis therapy (%)*		*Post ASA rhinitis therapy (%)*	
Nasal washes	3.8	Nasal washes	2.7
Nasal steroids	46.7	Nasal steroids	46.8
Antihistamines	2.9	Antihistamines	0.9
Combination	28.6	Combination	19.0
Unknown	18.0	Unknown	30.6

Types of reactions during desensitization were recorded and classified based on the data available (Table 4), as was medication used prior to and during desensitization (Table 5). Any adverse reactions to continued ASA use, and reasons for discontinuation of therapy were also assessed, and analyzed, as provided through chart review.

**Table 4:** **ASA data**

*History of ASA reaction (n, %)*	64 (61)
*Type of reaction (%)*	
Mixed symptoms*	2.1
Significant reaction**	14.9
Unknown	83.0

*Mixed (flushing, worsening nasal congestion, swelling).

**Significant reaction (anaphylactoid).

**Table 5:** **ASA Desensitization**

***Total desensitized (n = 111)***	
*Treatment during desensitization (n, %)*	
Yes	39 (35.1)
No	66 (59.5)
Unknown	6 (5.4)
*Treatment medication (n)*	
Nasal Steroid	2
Oral Steroid	21
Puffer (SABA, ICS, combination)	13
Epinephrine	1
*Patient improvement on ASA (n, %)*	
Yes	81 (73.0)
No	23 (20.7)
Unknown	7 (6.3)
*Patient improvement score (out of 3)*	
Change in taste or smell (1 pt)	34/111
Change in upper respiratory symptoms (1 pt)	78/111
Change in lower respiratory symptoms (1 pt)	67/111
0 out of 3	17 (15.3)
1 out of 3	20 (18.1)
2 out of 3	45 (40.5)
3 out of 3	24 (21.6)
Unknown	5 (4.5)

### Outcomes assessment

A priori rules were used regarding clinical benefit of ASA desensitization through patient statements, clinical notes, and imaging. Although ideal to compare imaging pre- and post- desensitization, the time interval and completeness of each patient was variable, and/or confounded by repeated surgical intervention or patient withdrawal. These data will be collected for a future, prospective study. Initial CT imaging was scored via the validated Lund-MacKay CT score (Table 2).

### Assessment of safety

Given the potential for significant reactions in a patient with known sensitivity to ASA and/or NSAIDs, the desensitization process was done in a controlled setting in hospital, with medications, including epinephrine close at hand. It was carried out using a validated protocol [3], conducted in a step-wise approach, with safety as a priority. Subjects were monitored closely by the physicians and nurses. Vital signs and physical examinations were taken, at a minimum, before and after each interval dose. Subjects were monitored for at least 1 hour after last dose of the day.

### Statistical analysis

Statistical analysis was performed using SAS 9.3 (SAS Institute, Cary, NC) and through Microsoft Excel™, including assessment of patient demographics and percentage calculations.

## Results

### Patient characteristics

The median age was 50.7 (17–75), and the population was split between males at 52.2% (n = 58) and females at 47.8% (n = 53). Most of the population had upper respiratory disease (77.8%, n = 86) and lower respiratory disease (66.3%, n = 74) for over 5 years. Based on limited retrospective data, it was difficult to assess baseline FEV1 severity and to assess for significant change during ASA desensitization.

In regards to baseline therapy, 80.8% (n = 90) were on a combination therapy for asthma, and 46.7% (n = 52) were on nasal steroids for rhinitis symptoms. After desensitization, there were comments in the patients record about being on less therapy, and the data trended towards decreased upper and lower respiratory therapy once on maintenance ASA therapy, but this did not reach statistical significance. As per the validated protocol [3], patients were started on 40 mg and titrated up to 162 mg on day one. On the second day, they were titrated up to 325 mg. From there the maintenance dose was 325 mg or 650 mg twice daily (Table 6).

**Table 6:** **ASA Maintenance therapy, need for resensitization, and sinus surgery during therapy**

**ASA maintenance**	Dose (mg)*	**BID (n, %)**	**TID (n, %)**
	325	37/80 (46.3%)	6/80 (7.5%)
	650	37/80 (46.3.)	0/80 (0.0%)
Patients resensitized (n, %)**		30/111 (27.0%)	
**Sinus surgery during desensitization**	**1** ^**st**^ **year (n, %)**	**2** ^**nd**^ **year (n, %)**	**3** ^**rd**^ **year (n, %)**
	15/111 (13.5%)	5/80 (6.3%)	6/80 (7.5%)

Note:

- N = 80 includes all those who achieved desensitization up to 1 year, with 10 lost to follow up, but kept as ‘intention to treat’.

- N = 111, includes all those who initially achieved desensitization.

*Reasons for ASA dose variations included: adverse effects, up- or down-titrated to symptoms or lack thereof, and/or interruption for surgery or illness.

**Reasons for resensitization included: restart post infection, sinus surgery, other surgery, and/or compliance.

In terms of AERD features, overall this population trended towards having more moderate-severe disease. Sixty-four percent (n = 71) required oral steroid therapy within the 12 months prior to desensitization therapy and 22.9% (n = 25) required antibiotics. The majority of the population, as seen in Table 2, had undergone more than one endoscopic sinus surgery, and most showed severe sinus of disease on imaging. This was confirmed by the severity in the Lund-MacKay score prior to desensitization with 97.4% (n = 108) scored ≥13 points.

### Outcomes assessment

Sixty-one percent (n = 68) had recorded previous reactions to ASA and/or NSAIDs exposure (Table 4).

Of those desensitized, 35.1% (n = 39) received pre-treatment or required treatment during desensitization, most commonly the use of an oral steroid (21/39 persons). One patient required epinephrine during the procedure, but was able to continue with the planned day two protocol and was successful in achieving desensitization and eventual improvement in symptoms.

Of those who achieved maintenance therapy, 73% (n = 81) stated improvement in symptoms of AERD. We created a patient improvement score with 1 point for improvement in taste or smell, 1 point for improvement in upper respiratory symptoms, and 1 point for improvement in lower respiratory symptoms for a total out of 3. Fifteen percent (n = 17) had no benefit, 18.1% (n = 20) had improvement in only one area, 40.5% (n = 45) had improvement in two areas, and 21.6% (n = 24) had improvement in all three.

### Safety assessment

In other published studies, known adverse effects occurred during the desensitization process (chest symptoms, worsened nasal congestion, rhinorrhea, facial flushing). In our study, one patient developed an anaphylactic reaction requiring epinephrine and prednisone. However, when re-challenged the patient on day 2 and they tolerated the dose of ASA, and did not require further rescue therapy. There has been anecdotal evidence that the more significant the adverse reaction, the more likely the subject is to have clinically significant improvement with desensitization, however, no study has specifically assessed this.

Of those who initially tolerated ASA maintenance therapy, 26.1% (n = 29) eventually developed adverse reactions (Table 7). Most common was gastrointestinal upset (n = 23). Of this group, 8 patients had a history of gastrointestinal reflux, on a proton-pump inhibitor, and 3 patients required the addition of a proton-pump inhibitor. Three patients had issues with easy bruising.

**Table 7:** **Adverse reactions and discontinuation of ASA**

*Adverse reactions (n, %)*	
Yes	29 (26.1)
No	71 (64.0)
Unknown	11 (9.9)
*Reactions (n)*	
Tinnitus	1
Gout	1
Gastrointestinal upset	23
Hypertension	1
Bruising	3
*Discontinuation of ASA therapy*	
Yes*	31 (27.9)
No	70 (63.1)
Unknown	10 (9.0)

*Most common reasons for discontinuation: patient belief not working, compliance, worsening symptoms, infection, surgery, and/or adverse effects.

Twelve months after desensitization, 27.9% (n = 31) had discontinued therapy. The most common reasons cited included lack of patient-perceived benefit, lack of compliance and thus subsequent need for repeat desensitization, worsened respiratory symptoms, or intolerable side-effects related to high-dose ASA.

## Discussion

This retrospective analysis looked at 111 patients who underwent ASA desensitization for AERD, who were followed for a maintenance period of approximately 12 months.

In regards to the first objective, of those who achieved maintenance therapy, 73% (n = 81) claimed symptom improvement. There was improvement noted in the chart review of overall symptoms, quality of life, and reduced need for rescue therapy for upper and lower respiratory symptoms. Patients were particularly impressed with any return of their sense of smell and/or taste. Using a score out of 3, relating to improved AERD symptoms, 15.3% (n = 17) identified no benefit, however, the remaining 94 patients had some form of improvement, with 21.6% (n = 24) having had an improvement in all 3 areas of sense of taste/smell, upper, and lower respiratory symptoms.

These values are similar to those seen in the literature. A retrospective study by Berges-Gimeno et al. [8] of 172 patients found a statistically significant improvement in the number of sinus infections, ability to smell, and upper and lower respiratory symptoms after one year of maintenance ASA therapy. There were also fewer hospitalizations for asthma, and a reduction in the use of nasal, inhaled and oral corticosteroids. Overall, 87% were said to have responded to ASA therapy.

A randomized, double blind study by Swierczynska-Krepa M et al. [14] found improved smell, peak nasal inspiratory flow, and quality of life amongst aspirin-intolerant asthma patients on ASA maintenance.

A randomized controlled trial by Lee et al. [13], took 137 patients randomized to receive ASA 325 mg or 625 mg twice daily. Then after 1 month the group either increased or decreased their dosage based on symptoms. There was a statistically significant improvement in sinus infections and operations, hospitalizations for asthma, and upper and lower respiratory symptoms. After one year, there was a statistically significant reduction in intranasal and oral corticosteroid use.

In our study, patients were mostly on ASA 325 mg or 650 mg twice daily (Table 6). Overall, 46.3% were on 325 mg twice daily, and 46.3% were taking 650 mg twice daily. Six patients (7.5%) were on 325 mg three times daily, as they were in-between dosage adjustments. Dosage adjustments varied based on a multitude of factors such as adequate or inadequate symptom control, adverse effects, compliance, and interruptions for surgery or illness.

It is difficult to say that the ideal dose is, and the literature has shown benefit for both regimens [3,5,13]. As such, it becomes an area of individualized therapy based on patient response.

Overall this study had a patient population with severe AERD, and many with a history of multiple nasal surgeries (Table 2). The literature has shown that ASA desensitization has resulted in improved nasal polyposis and less nasal surgeries [5,15]. A recent study by Cho et al. [16] demonstrated a sustained improvement in endoscopic and symptomatic nasal polyposis symptoms of those on ASA maintenance therapy.

In our study, fifteen patients (13.5%) underwent nasal surgery within the first year of desensitization. Then 6.3% and 7.5% underwent nasal surgery within the second and third years of desensitization, respectively (Table 6).

In our study, at the end of 12 months, 27.9%, (n = 31) had discontinued therapy. Two of those patients were asked to discontinue therapy for upcoming surgery. Another two stopped due to infection, with the inability to maintain therapy. Otherwise, the rest who discontinued therapy claimed lack of perceived benefit, issues with compliance of high-dose ASA twice a day, worsened AERD symptoms, or adverse effects related to ASA therapy.

Among those who tolerated ASA therapy, 26.1% (n = 29) had adverse events that were likely due to the ASA. Most commonly this was gastrointestinal upset, and a few patients needed to be started on proton-pump-inhibitor therapy. Three patients had issues with easy bruising, 1 with tinnitus, 1 patient developed gout, and 1 patient had worsened hypertension. There were no life-threatening adverse reactions during the 12-month follow up. This is a similar to past literature where 10-50% of patients discontinue, and 20-30% complain about gastritis and reflux symptoms [3,6,8].

The retrospective study by Berges-Gimeno et al. [8] showed that 13% discontinued, with 67% of that group having gastrointestinal symptoms. Of the 172 patients, 11% failed to respond to therapy.

From the randomized study by Lee et al. [13], 23.4% discontinued due to adverse effects, with 37.5% of that group having dyspepsia.

Limitations in this study include those associated with any retrospective study and analysis, particularly lack of data points, such as pre- and post- FEV1 percent-predicted values. A randomized controlled trial would have been the preferred method. Additionally, not all patients were initially challenged to prove ASA sensitivity, rather the focus was on clinical history. Dosing was carried out by different physicians and not controlled for. In assessing response to ASA maintenance therapy, there was no objective and validated scoring system used. Lastly, follow-up was only 12 months, and it would have been worthwhile following up this population longitudinally to assess benefit-risk ratios for clinical outcomes versus adverse effects. Pre- and post- CT sinus imaging may have provided a better diagnostic assessment of the effectiveness of desensitization therapy, however post-therapy imaging is not the current standard of care, nor is there sufficient data to correlate the association between symptoms and imaging on post-therapy images.

With respect to future directions, a prospective, randomized controlled study, would be beneficial to gather key clinical data points, as well, to assess baselines characteristics, prove ASA sensitivity, and determine if there are certain clinical predictors that indicate which patients would be better candidates for ASA desensitization. In previous studies, those who were able to maintain a high-dose ASA regimen successfully were more likely to be: less than 40 years old, a poor sense of smell, multiple prior respiratory reactions, or to have had severe prior asthmatic reactions associated with aspirin and NSAIDs [15]. However, to our knowledge, there is no published literature on validated and reproducible patient predictors of clinical benefit from ASA desensitization.

## Conclusion

This study assessing 111 patients who underwent ASA desensitization for AERD showed overall effectiveness, with 81/111 patients claiming improvement in symptoms and 31/111 discontinuing maintenance ASA therapy. This is the first Canadian study assessing ASA desensitization and maintenance therapy in the AERD population. A validated protocol is in place, and many centres outside of Canada have significant reproducible data showing benefit to ASA desensitization in this population.

The ability to desensitize the patient to ASA not only aids in symptom improvement and a gain in quality of life in this population, but it also allows the use of ASA and NSAIDs in patient populations who require these medications for other reasons. We believe ASA desensitization should be considered and performed more frequently in the appropriate clinical situations in Canada.
